# 3D printing fluorescent material with tunable optical properties

**DOI:** 10.1038/s41598-021-96496-0

**Published:** 2021-08-24

**Authors:** Alberto J. Ruiz, Sadhya Garg, Samuel S. Streeter, Mia K. Giallorenzi, Ethan P. M. LaRochelle, Kimberley S. Samkoe, Brian W. Pogue

**Affiliations:** 1 Thayer School of Engineering, Dartmouth College, 14 Engineering Dr., Hanover, NH 03755 USA; 2 QUEL Imaging LLC, 85 N Main Streeet, White River Junction, VT 05001 USA

**Keywords:** Biomedical engineering, Imaging and sensing, Biomarkers

## Abstract

The 3D printing of fluorescent materials could help develop, validate, and translate imaging technologies, including systems for fluorescence-guided surgery. Despite advances in 3D printing techniques for optical targets, no comprehensive method has been demonstrated for the simultaneous incorporation of fluorophores and fine-tuning of absorption and scattering properties. Here, we introduce a photopolymer-based 3D printing method for manufacturing fluorescent material with tunable optical properties. The results demonstrate the ability to 3D print various individual fluorophores at reasonably high fluorescence yields, including IR-125, quantum dots, methylene blue, and rhodamine 590. Furthermore, tuning of the absorption and reduced scattering coefficients is demonstrated within the relevant mamalian soft tissue coefficient ranges of 0.005–0.05 mm^−1^ and 0.2–1.5 mm^−1^, respectively. Fabrication of fluorophore-doped biomimicking and complex geometric structures validated the ability to print feature sizes less than 200 μm. The presented methods and optical characterization techniques provide the foundation for the manufacturing of solid 3D printed fluorescent structures, with direct relevance to biomedical optics and the broad adoption of fast manufacturing methods in fluorescence imaging.

## Introduction

Fluorescence imaging for surgical guidance is a modality that provides real-time contrast enhancement of key medical biomarkers in the operating field for applications such as tissue perfusion and cancer margin detection^1,2^. Given the growth of fluorescence-guided surgery (FGS) in a range of surgical indications with an increasing number of devices, there is a compelling need for fluorescent imaging targets that enable system characterization, performance monitoring, algorithm development, and inter-system comparisons^3–5^. Despite this need, no imaging targets for FGS have been widely adopted or made commercially available. This is partly due to challenges related to manufacturing scalability, application-specific optical spectra, and long-term stability of the imaging targets^6–11^. 3D printing of fluorescent materials with tunable optical properties has been developed in this study as a tool to address these challenges.

The last 2 decades have seen continued development in the design and manufacturing of imaging targets and phantoms for biomedical optics. A general design trade-off exists between mimicking biochemical parameters (e.g., using intralipid as a scatterer) and long-term stability (e.g., using TiO_2_ as a scatterer), where the latter is favored for enabling widespread adoption of targets^3,5^. Creating stable imaging targets requires addressing both mechanical and photostability issues. To simultaneously tune optical properties, incorporate fluorophores, and provide mechanical stability, epoxy^12,13^ and polyurethane^11,14–17^ have been widely used as base materials; however, the associated manufacturing complexity, casting methodology, and limited tuning of rigidity are drawbacks. To address photostability issues linked to organic fluorophores, quantum dots^16,18,19^ and doped-polymers^20^ have been proposed, both of which suffer from the limited tuning of the application-specific spectra and biological relevance. In recent years, 3D printed (3DP) biomedical optics phantoms have shown the ability to mimic anatomical structures and streamline the manufacturing process, with the main drawback of providing only limited tuning of absorption and scattering properties^6–10^. Despite these advances in 3D printing, no comprehensive method for simultaneous incorporation of fluorophores and fine-tuning of absorption and scattering properties has been presented. Existing high-temperature deposition of fused fabrication techniques^8,21^ can cause fluorophore degradation while proprietary photopolymer-based printing systems, including stereolithography (SLA)^9,10^ and material jetting techniques^6,7,11^, have limited the tuning of optical properties and printing parameters.

In this study, we introduce a method for manufacturing 3DP fluorescent material and demonstrate the incorporation of various fluorophores, including organic dyes and quantum dots, alongside the tuning of optical absorption and scattering properties. The developed method utilizes photopolymer-based 3D printing techniques with commercially available resins and liquid crystal display (LCD) masked stereolithography (MSLA) 3D printers to adjust the printing parameters and enable cost-effective manufacturing. This approach creates a doped 3DP polymer matrix by incorporating pre-dispersed fluorophores, scatterers, and absorbers that are printed via the adjustment of photopolymerization light dosage and layer height resolution. Optical characterization was performed on 3DP solid cuvettes and blocks of material used to measure the transmission spectra, fluorescence spectra, and coefficients of absorption and reduced scattering. Using this method, we manufactured biomimicking and complex geometric structures with feature sizes less than 200 μm that match the spectral behavior of indocyanine green (ICG), the most widely used fluorescent contrast marker in FGS^1,22^. Optical characterization of various resins and 3DP fluorescent materials demonstrated the potential spectral shifts and relative fluorescence emission yield changes associated with this methodology. Furthermore, tuning of absorption and reduced scattering coefficients of 3DP material is demonstrated within the relevant soft tissue coefficient ranges of 0.005–0.05 mm^−1^ and 0.2–1.5 mm^−1^^23^, respectively. The presented methods and optical characterization techniques set the foundation for the manufacturing of 3DP fluorescent materials, with direct relevance to biomedical optics and the broad adoption of fluorescent imaging targets.

## Results

### 3D printable fluorescent resin with tunable optical properties

Photocurable resins used in 3D printing are generally composed of monomers and oligomers, (meth)acrylites, and photoinitiators, along with other additives such as UV blockers, pigments, and dispersion agents^24^. In commercially available resins, the composition and ratio of these components are optimized to achieve specific mechanical properties and tune photo-reactivity based on the 3D printed technique used. Resins for LCD MSLA 3D printers typically contain higher photoinitiator concentrations to account for lower curing irradiances when compared to SLA and digital light projection (DLP) printing techniques.

**Figure 1:**
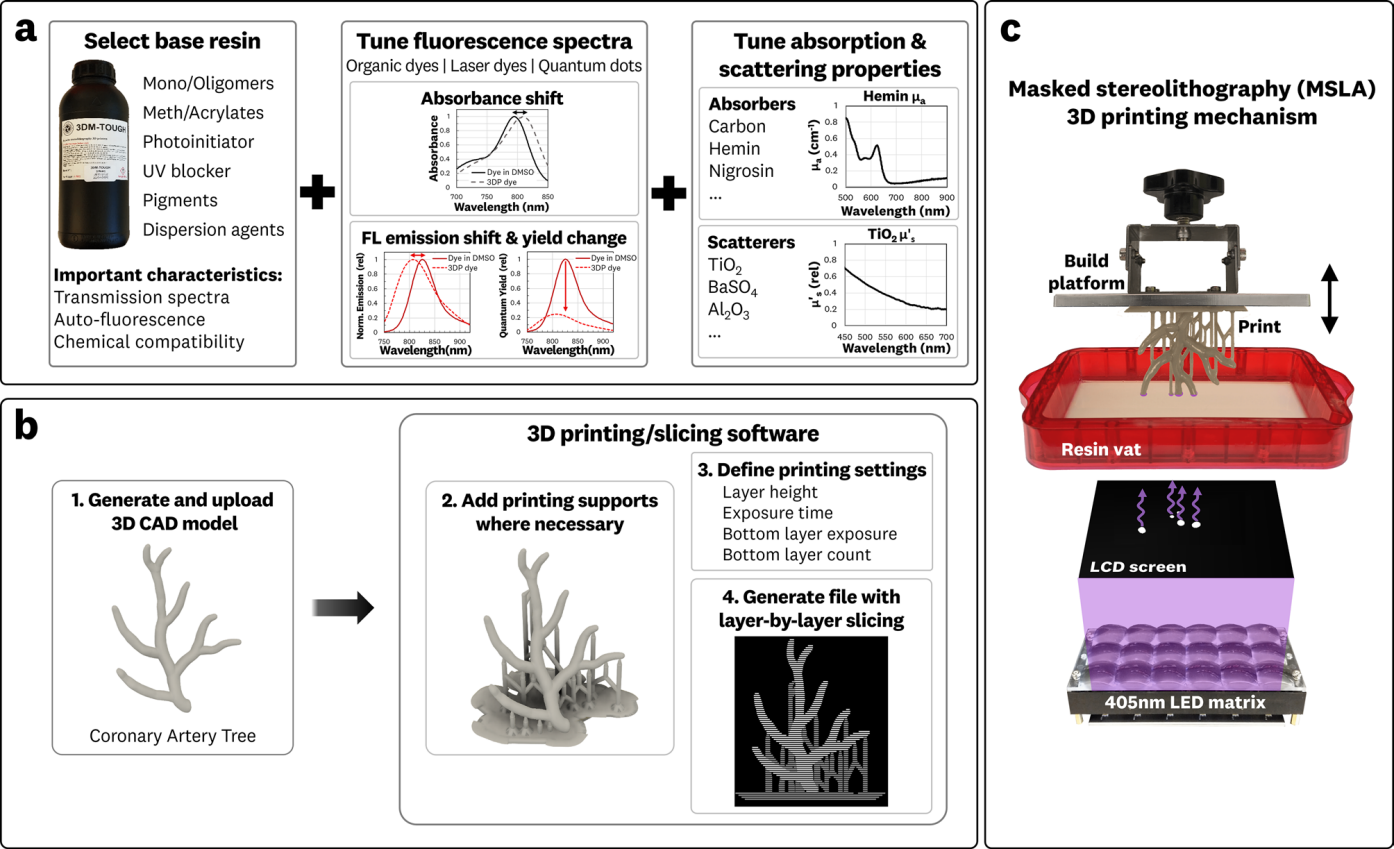
Preparation and 3D printing of fluorescent material with tunable optical properties using LCD-based masked stereolithography printing. (**a**) Tuning of application-specific fluorescence, absorbance, and scattering properties was achieved through the addition of pre-dispersed solutions of fluorescent dyes, absorbers, and scatterers. (**b**) 3D model of a coronary artery created from an MRI scan^25^. This model was prepared for printing by adding mechanical supports, defining printing settings for the specific resin and 3D model, and generating a layer-by-layer printing file. (**c**) The masked stereolithography 3D printing mechanism was used where the LCD screen selectively exposes the resin vat, layer-by-layer, as per the printing file generated in (**b**) resulting in a 3DP structure.

Figure 1 shows an overview of the entire 3D printing methodology. The process of creating fluorescent photocurable resins with tunable optical properties involved the selection of a base photopolymer resin and incorporation of pre-dispersed fluorophores, absorbers, and scatterers (Fig. 1a and Supplementary Fig. 1). Clear, unpigmented formulations of commercially available resins designed for LCD MSLA and DLP 3D printers with a curing wavelength of 405 nm were used. The pre-dispersion methods utilized here were adapted from techniques developed in the manufacturing of polyurethane-based phantoms^14,15,17^, where stock solutions of the additives were prepared in dimethyl sulfide (DMSO) as an intermediary step to aid in particle dispersion and result in a homogeneous resin solution. These stock solutions can be manufactured at different concentrations and added at different ratios to tune the fluorescence, absorption, and scattering properties of the 3DP resin. As a rule of thumb, the weight of the added stock solutions was kept to < 5% of the resin weight to prevent printing problems associated with dilution of the photopolymerization formulation. The prepared resin could be stored at room temperature, but resuspension of the additives through agitation and sonication would be required.

### 3D printing of fluorescent and optically tunable material

Alongside the fluorescent resin preparation, a 3D model and associated printing file are generated to provide the layer-by-layer instructions to the printer (Fig. 1b). The MSLA printer (Fig. 1c) uses an inverted lithography set-up with a 405 nm LED matrix and LCD screen to provide selective voxel curing that results in the finished 3DP structure (Supplementary Movie 1).

The 3D models, for example, an MRI scanned artery tree^25^, were prepared for printing by creating the necessary mechanical supports, which are recommended for structural overhangs exceeding 45°, and bottom raft to provide adhesion onto the build platform. The associated printing files were generated using commercially available software. The single-layer curing dose (exposure) and layer height parameters were set for the specific 3D model and fluorescent resin formulation. The ability of the LCD MSLA mechanism to easily tune the curing dose and layer height enabled the printing of complex structures and custom formulations of fluorescent resin.

Generally, the addition of absorbers and scatterers increases the resin’s effective attenuation coefficient, requiring an increased photopolymerization curing dose compared to the unpigmented resin base^24,26,27^. Increasing single layer exposure times and reducing the printing layer height are straightforward ways to achieve printability in the modified resins. Exposure times and layer height are tuned through the 3D slicing software (Fig. 1b), with typical values ranging between 2.0–15.0 s and 0.01–0.10 mm respectively.

Given the inverted stereolithography printing mechanism, the forces associated with separating a newly printed layer from the resin vat must be considered to prevent detachment of the 3DP structures from the build platform. To avoid detachment during printing, the initial layers (the first ~ 300 μm) needed to have a 3–5 × longer curing exposure and a larger cross-sectional area than the subsequent layers; this ensured strong adhesion to the build platform. To limit potential overheating problems of the LED matrix and LCD screen degradation, exposure times were limited to ~ 60 s and 15 s or less for the first layers and subsequent layers, respectively. The resin’s photoinitiator concentration was increased to reduce the required exposure time whenever the single-layer photopolymerization was not achieved within a 15 s layer exposure.

A cleaning bath and post-curing process followed the layer-by-layer selective voxel crosslinking to remove excess uncured resin and finalize the photopolymerization of the 3DP structure. The printed parts were cleaned using an isopropyl alcohol bath and then allowed to dry. The post-curing process involved exposure of the print to low-irradiance 405 nm light to finalize curing, especially on the surface of the prints.

**Figure 2:**
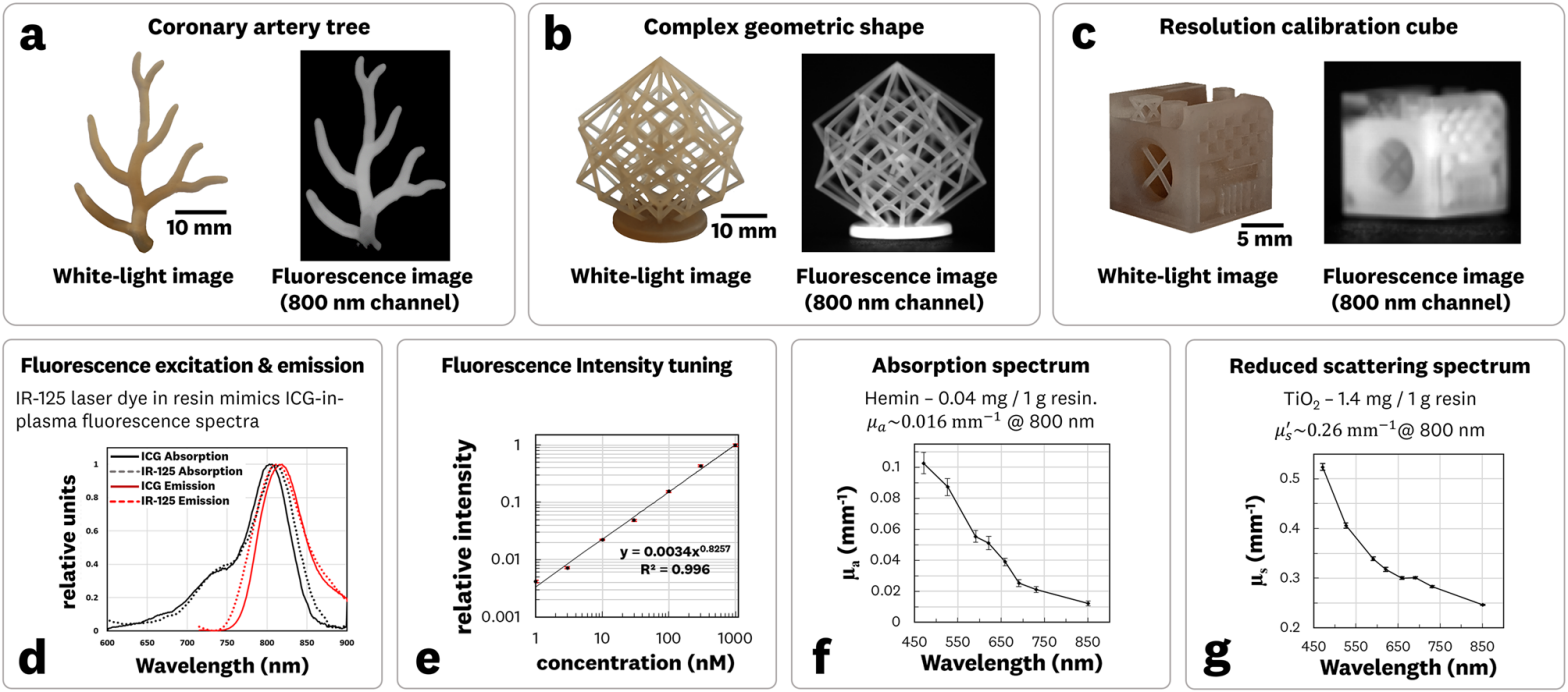
3D printed ICG-equivalent material with tuned optical properties. (**a–c**) White-light and 800 nm channel fluorescence images of (**a**) MRI-scanned coronary artery tree structure, (**b**) complex geometrical lattice cube, and (**c**) resolution calibration cube with sub-200 μm feature sizes. (**d**) Fluorescence emission and excitation spectra of 3DP fluorescent material and ICG-in plasma showing close overlap in spectral features. (**e**) Linearity of fluorescent intensity output for increasing laser-dye concentrations in 3DP material. (**f, g**) Measured absorption and reduced scattering coefficient spectra of the 3DP material. Error bars indicate ± one standard deviation from the mean.

Using the described method, 3DP structures that mimic ICG fluorescence, tissue absorption, and tissue reduced scattering properties were manufactured (Fig. 2). The prepared resin integrated IR-125 laser dye, hemin, and TiO_2_ as the fluorophore, absorber, and scatterers, respectively. The MRI scanned artery tree (Fig. 2a) demonstrated the ability to print anatomy mimicking structures. The complex geometric shape (Fig. 2b) showcased the printing of interweaving, self-supporting structures. The resolution calibration cube (Fig. 2c) confirmed the ability to print feature sizes less than 200 μm. Fluorescence images (Fig. 2a–c) and spectra measurements (Fig. 2d) of the 3DP material showed good correlation to the spectra and fluorescence emission of ICG-in-plasma; this demonstrates the ability to 3D print material that is spectrally equivalent to the most used contrast dye in FGS.

The measured relative spectral shifts between the IR-125 doped 3DP material and ICG-in-plasma were a 5 nm red shift for absorption and 2 nm blue shift for the emission. The material was printed at varying IR-125 concentrations (1–1000 nM) to demonstrate the ability to tune fluorescence intensity, resulting in a power fit with R^2^ = 0.996 (Fig. 2e). The absorbing and reduced scattering coefficients were measured as μ_a_ = 0.016 mm^−1^ and μ_s_′ = 0.26 mm^−1^ at 800 nm, corresponding to concentrations of 0.04 mg/g and 1.4 mg/g of hemin and TiO_2_ to resin, respectively.

In the following sections, the optical properties of the 3DP materials are explored further, with emphasis on determining (1) base resin spectral characteristics including the transmission and autofluorescence shifts associated with the post-curing process, (2) absorption and fluorescence spectra emission shifts and relative emission yield changes associated with the printing methodology, (3) fluorescence photostability, and (4) tuning of the absorption and scattering properties of the 3DP material.

### Optical properties of photocurable resins

**Figure 3:**
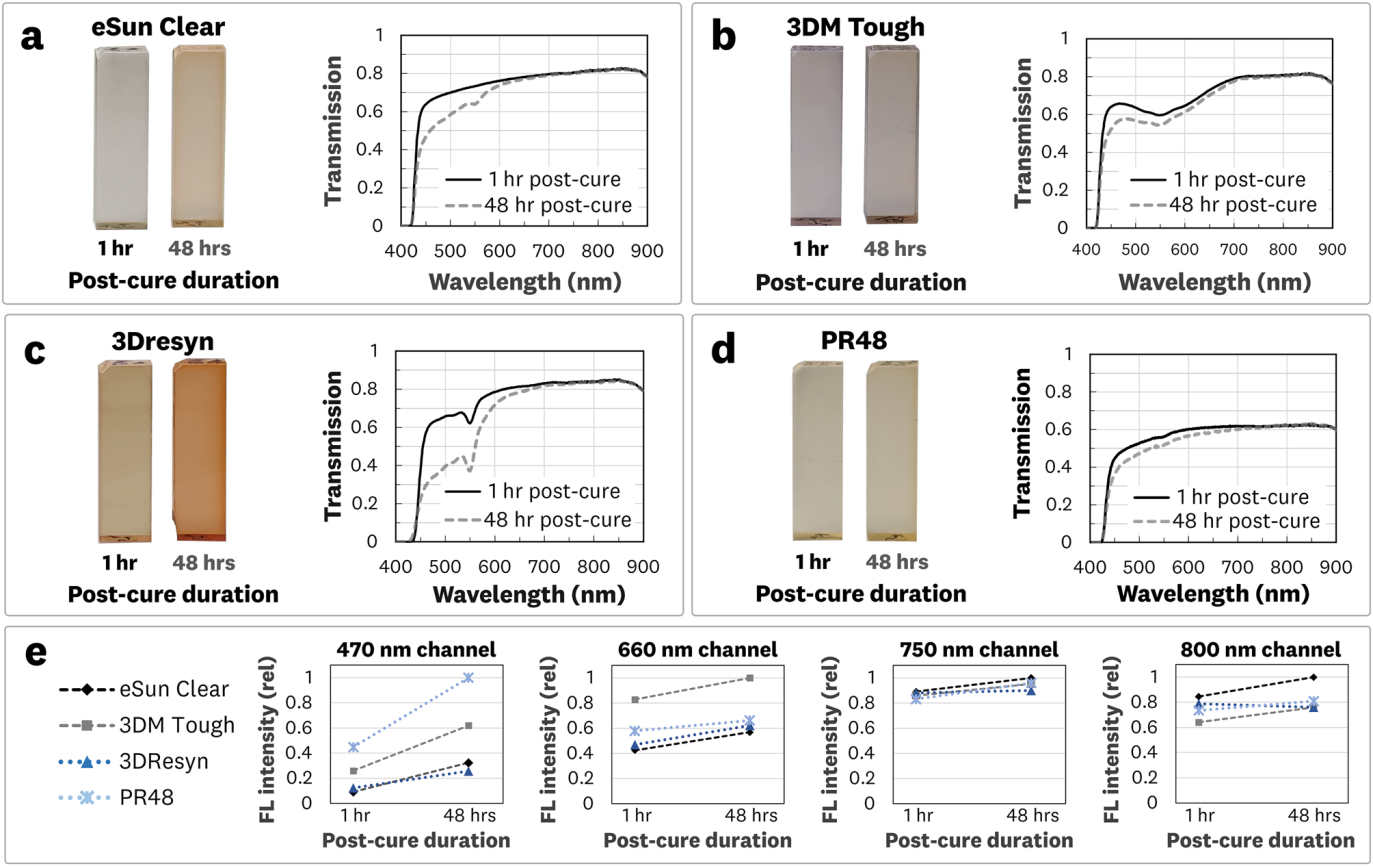
Optical transmission and autofluorescence measurements of 3D printed photocurable resins. Measurements are performed on 3DP solid cuvettes (12 × 12 × 40 mm^3^) of four commercially available clear resins that were post-cured for 1 h and 48 h. (**a**–**d**) Transmission images and spectra. (**e**) Relative autofluorescence measurements.

Four commercially available resins, designed for LCD MSLA and DLP 3D printers with a curing wavelength of 405 nm, were characterized to understand the optical properties of photocurable polymers. The transmission spectra, autofluorescence, and the relative changes associated with the post-curing process are examined in this section 3DP solid cuvettes of the resins were optically polished to provide accurate spectral absorbance and emission measurements (Supplementary Fig. 2). Figure 3 shows the results of the transmission and autofluorescence measurements for the 3DP cuvettes.

It is well known that the post-curing of photocurable resins can lead to a visual “yellowing” of 3DP structures^28^. To study the effect of post-curing on the 3DP material’s optical properties we provided low-intensity 405 nm irradiation for 1 h and 48 h followed by measurement of the transmission spectra (Fig. 3a–d) and quantification of the material autofluorescence at various excitation wavelengths (Fig. 3e).

The measured transmission spectra for the 3DP cuvettes shows varying baseline transmission and spectral shifts in the visible wavelengths that account for the observed “yellowing” associated with the post-curing procedure. All resins showed low transmission below the 450 nm wavelength given their inherent formulation for curing at 405 nm. This indicates that the method is limited to fluorophores with excitation above ~ 450 nm. Furthermore, all four resins exhibited relative flatness and minimal shift of the transmission spectra in the 700–900 nm range.

The autofluorescence measurements showed an increasing signal trend for the 48 h post-cured samples for all printed resins. The autofluorescence values varied between resins and between fluorescence channels, emphasizing the need to characterize post-processed material under expected imaging conditions. The autofluorescence from base resins could be considered as “background” signal and is best compared to the fluorescence signal obtained from fluorophore-doped photopolymers. For example, the measured background autofluorescence for all the resins in the 800 nm imaging channel resulted in measured signals equivalent to < 0.1 nM of IR-125 doped 3DP material. For reference, this signal is below the measurement sensitivity levels of previously characterized FDA-approved ICG-specific imaging systems^11^.

These results indicate that the absorbance spectra, autofluorescence, and shifts in the transmission spectra associated with the post-curing process should be considered when selecting a base resin for a given application.

### Fluorescence spectra shift and emission yield change

**Figure 4:**
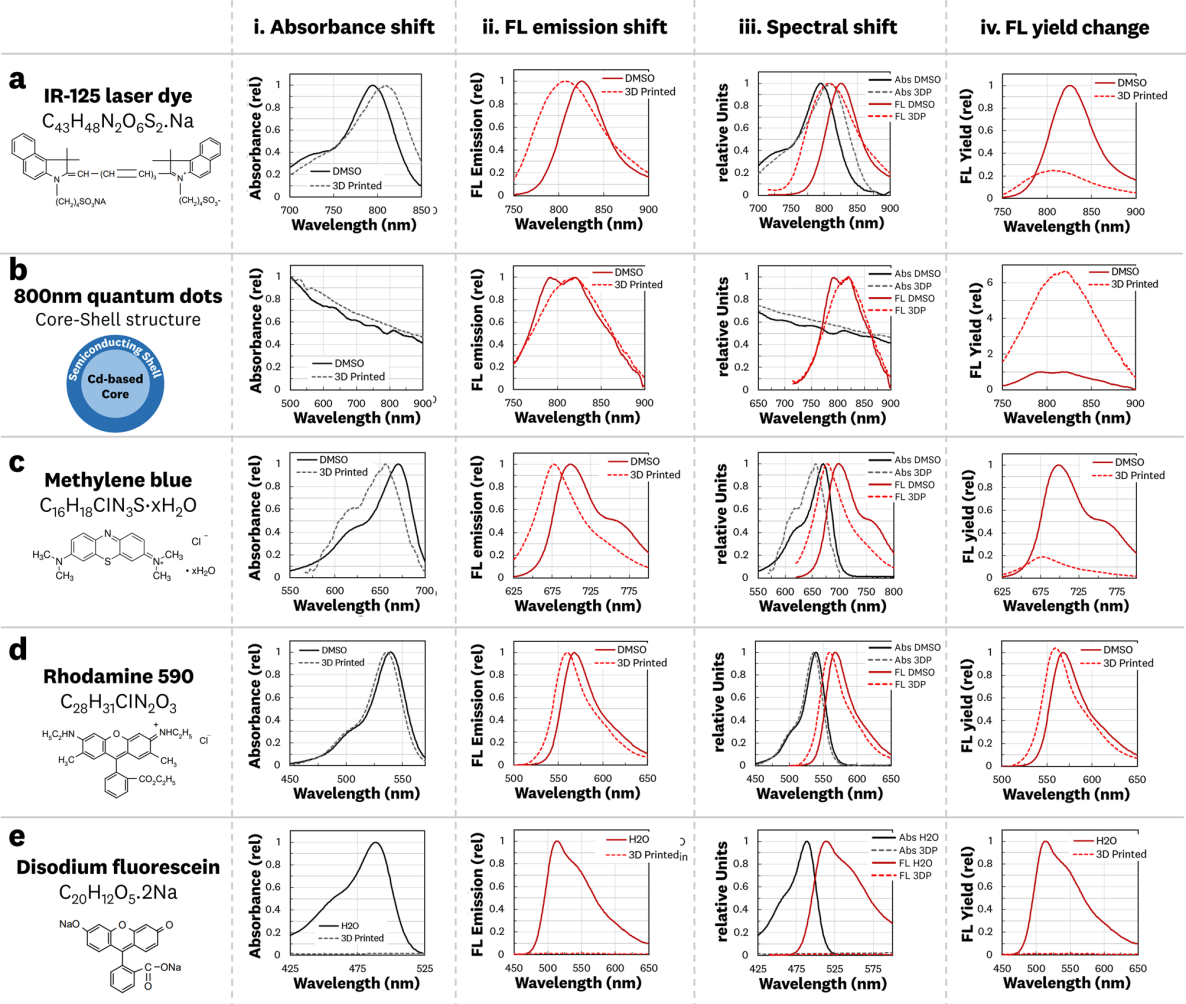
Spectral shifts (**i–iii**) and relative fluorescence yield change (**iv**) for 3D printed fluorescent materials compared to in-solvent fluorophores. (**a**) IR-125 laser dye shows significant spectra shift and broadening with a 0.24 × relative fluorescence yield. (**b**) 800 nm quantum dots show small shifts in spectra with a 6.7 × relative fluorescence yield. (**c**) Methylene blue shows a significant shift in spectra with a 0.18 × relative fluorescence yield. (**d**) Rhodamine 590 laser dye shows small spectra shifts and no significant change in the fluorescence yield. (**e**) Disodium fluorescein dye is inactivated in the 3D printing process, showing no detectable absorbance or emission spectra.

Tuning of the resin’s fluorescent properties was achieved by integrating pre-suspended organic dyes, laser dyes, and quantum dots. The main concern of incorporating fluorophores into 3DP resin is changes in the fluorescence spectra and emission yield. We confirmed the ability to 3D print various fluorophores through incorporation and spectral characterization of IR-125, 800 nm quantum dots, methylene blue, rhodamine 590, and disodium fluorescein. Fluorescence spectra measurements were performed on fluorophore-doped 3DP solid cuvettes that were post-cured for 1 h and optically polished. Equivalent concentration solutions of the fluorophores in DMSO (H_2_O for disodium fluorescein) were used as “reference” standards for calculating spectral shifts and emission yield changes. Figure 4 shows the measured spectral shifts and emission yield changes for these fluorophores.

The IR-125 laser dye (Fig. 4a) was printed at 1000 nM and showed a 14 nm red absorbance shift and 18 nm blue emission shift with a widening of the spectral features and a 0.24 × relative emission yield. The 800 nm cadmium-based quantum dots (Fig. 4b) were printed at 10 nM showing no significant changes in spectral features and a 6.7 × relative emission yield. Methylene blue (Fig. 4c) was printed at 10 μM, showing blue shifts of 14 nm for absorbance and 22 nm for emission with a 0.18 × relative emission yield. Rhodamine 590 (Fig. 4d) was printed at 10 μM, showing blue shifts of 3 nm for absorbance and 8 nm for emission with no significant change in the emission yield. Disodium fluorescein was printed at 10 μM, showing total fluorophore inactivation for the 3DP material. However, a fluorescence signal was detected for disodium fluorescein suspended in uncured resin, such that the cause of the loss of signal in the 3DP material was likely a combination of (1) photophysical inactivation in the fixed photopolymer matrix and (2) photobleaching from the high-irradiance 405 nm curing light due to the significant overlap with the absorption spectra of the fluorophore. The differences in spectral shifts and relative fluorescence yield changes for the 3DP materials emphasize the need to characterize an individual fluorophore’s performance to ensure compatibility with application-specific spectral requirements.

To further explore fluorescence spectra shifts, IR-125 doped material was printed with three commercially available resins. Fluorescence spectra measurements were performed for uncured resin and 3DP cuvettes that were post-cured for 1 h and 48 h (Supplementary Figs. 3 and 4). The spectral measurements show only minor differences in the fluorescence shifts between the three different resins. However, there is a significant spectral shift between uncured resin and the 3DP cuvettes, highlighting the need to characterize the 3DP material rather than the prepared resin for accurate optical measurements. There were minimal shifts (< 1 nm) in the spectral features between the 1 h and 48 h post-cured samples. Furthermore, fluorescence imaging at the 800 nm channel showed no significant difference in the collected fluorescence signal between the two different post-curing times. The observation of shifts in fluorescence spectra and emission yields during the 3D printing process match previously reported changes caused by localized viscosity of doped epoxy^29,30^ and photocurable resins^31^.

### Photostability of 3DP fluorescent material

Various samples of IR-125 doped photopolymer at varying concentrations were printed to test the 3DP material’s photostability. The 3DP samples underwent fluorescence imaging for 2 months (Supplementary Fig. 5), showing no significant decay of the fluorescence signal over this time period. These results are consistent with previously reported photostability measurements of the laser dye in solvent^32^ and in polyurethane^11,14^.

### Tuning of absorption and scattering coefficients

The fine-tuning of the absorption and reduced scattering coefficients was achieved by varying the concentration of the added pre-dispersed absorbers (i.e., carbon, hemin, nigrosin, etc.) and scatterers (i.e., TiO_2_, BaSO_4_, Al_2_O_3_, etc.). Since the photopolymerization process can alter optical properties, measurements of the absorption and reduced scattering coefficients were performed on the post-cured 3DP material.

**Figure 5:**
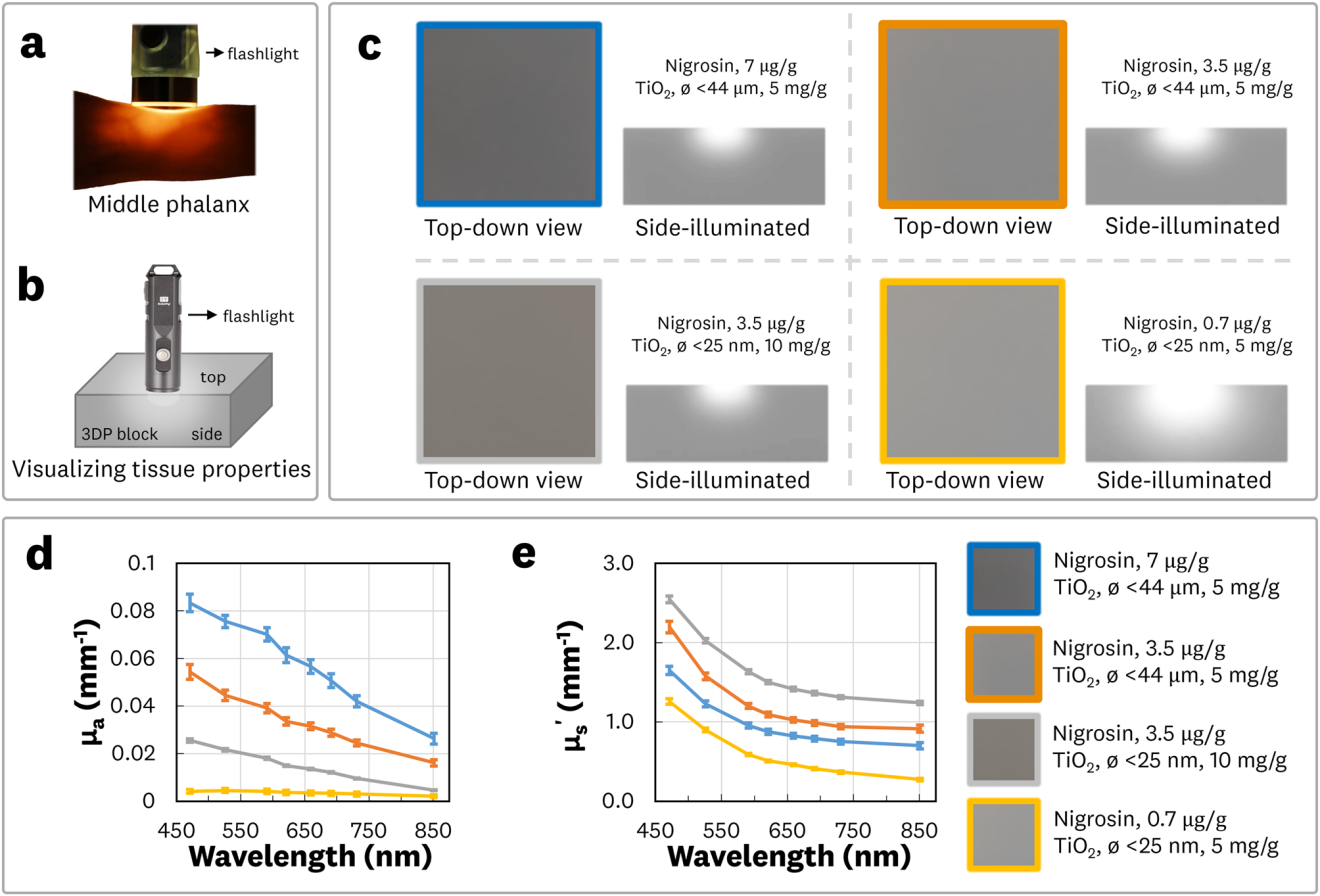
Visualization and measurement of absorption and reduced scattering properties. (**a**) Image of a side-illuminated middle phalanx. (**b**) Side-illuminated method for visualization of optical properties of 3DP 50 × 50 × 20 mm^3^ blocks. (**c**) Top-down and side-illuminated images of four 3DP blocks with varying nigrosin and TiO_2_ concentrations. Measured absorption (**d**) and reduced scatter (**e**) coefficients spectra for the four 3DP blocks. Error bars indicate ± one standard deviation from the mean.

To demonstrate the ability to tune the absorption and reduced scattering coefficients, blocks of material (50 × 50 × 20 mm^3^) with varying concentrations of nigrosin and TiO_2_ were printed. Visualization and measurement of the optical coefficients are shown in Fig. 5. Side illumination of a finger (Fig. 5a) and the 3DP blocks (Fig. 5b) allows for qualitative visualization of the optical properties. Four blocks with varying absorber and scatterer concentrations were imaged in a top-down and side-illuminated configuration with fixed exposure and gain (Fig. 5c) to visually compare the light propagation within the 3DP material. We performed multiwavelength spatial frequency domain imaging (SFDI) on the 3DP blocks to quantify the absorption and reduced scattering coefficients. Plots of the resulting deconvolved coefficient spectra (Fig. 5d, e) show tuning within the range of relevant soft tissue coefficients of 0.005–0.05 mm^−1^ and 0.2–1.5 mm^−1^ for absorption and reduced scattering, respectively^23^. The ability to 3D print material at different sizes and thicknesses could allow straightforward implementation of other characterization techniques for measurement of the optical coefficients. Furthermore, it is worth noting that two different particle sizes of TiO_2_ were used, showcasing the potential of modifying the anisotropy of scattering with the presented manufacturing methodology. Mathematical formulations for tuning the absorption and reduced scattering are outside the scope of this study, but methodologies presented for polyurethane phantoms could be adapted in future work^14^.

## Discussion

In summary, the presented method allowed for the manufacturing of 3D printed fluorescent material with tunable absorption and reduced scattering properties. The method relies on doping the photopolymer matrix of clear resins by incorporating pre-dispersed solutions of fluorophores, absorbers, and scatterers. The method uses commercially available photocurable resins and LCD MSLA 3D printers, allowing for accessible and cost-effective manufacturing of the 3DP material. The versatility of the LCD MSLA mechanism in tuning the light-curing dose and printing height parameters allowed for the manufacturing of complex 3D structures and custom resin formulations.

The optical characterization of the 3DP materials showed the relevant characteristics for manufacturing application-specific structures, including base resin transmission and autofluorescence, fluorescence spectral shifts, and tuning of absorption and scattering properties.

Absorbance measurements of base resins showed differences in baseline transmissions alongside spectral and autofluorescence shifts associated with the post-curing process. All the tested resins, formulated for 405 nm curing, showed relatively flat transmission and negligible shifts in the NIR spectra beyond 650 nm, indicating significant potential in manufacturing application-specific 3DP targets for NIR fluorescence applications.

Spectral characterization of various 3DP fluorophores, including organic dyes and quantum dots, showed the potential absorbance and fluorescence spectral shifts and emission yield changes associated with the methodology. Some dyes are incompatible with embedding in the photopolymer matrix, such as disodium fluorescein, whereas others such as Rhodamine 590 are largely unchanged in the 3D printing process. These photophysical results highlight the importance of performing spectral measurements on the post-processed 3DP material to account for spectral changes in the manufacturing process.

Long-term photostability tests of IR-125 doped 3DP material demonstrated the potential to manufacture stable imaging targets suitable for use in cross-system comparison and performance monitoring of FGS systems.

The presented method’s main drawback was the limitation of single material prints due to the vat polymerization technique used in LCD MSLA printers, where the 3DP structure is constructed within the vat of liquid photopolymer. Furthermore, as previously presented with polyurethane manufacturing methods^11^, the potential of altered environment/chemistry to the organic molecules may lead to unintended spectral shifts and photophysical fluorescence yield changes. As the results showed, each fluorophore requires careful characterization once embedded within the matrix material, and selection of compatible photopolymers for each dye is important. It is likely that hydrophilic dyes commonly used in biomedical applications are not ideal for 3D printing. Rather, mildly altered versions of the dyes that are more stable in organic solvents may be suitable to achieve matching optical properties. A good example of this is indocyanine green, which is a notoriously unstable dye, but has the analog of IR-125, which is sold as a laser dye, and is stable in the resin matrix. Similarly, rhodamine 590 is another laser dye, designed for use in organic solvents, and provides a nearly lossless fluorescence in the 3DP matrix when compared to a DMSO solution. However, for other dyes such as disodium fluorescein, the environment of the 3DP resin could render this molecule either aggregated or otherwise photophysically inactivated. Photobleaching through a significant absorption overlap with the curing wavelength can also be a primary mechanism of fluorophore inactivation during the photopolymerization procedure.

Although this was not observed for the printed structures in this study, it is worth noting that precipitation of additives during the printing procedure can lead to shifts in the optical properties of 3D printed structures along the z-axis. To minimize this effect, resin should be mixed immediately before manufacturing and printing times minimized (< 1 h total printing time was generally used for the 3DP structures). Exploration into dispersion agents, in-print resin mixing, and additive precipitation rates would be warranted for structures that require extended printing times or resin formulations that have a high concentration of additives.

Future studies could include implementing the presented method into material jetting systems to enable multi-material printing of fluorescent structures, the development of scattering anisotropy tuning using scatterers with varying particle sizes, and the printing with resins that mimic the mechanical properties of soft tissue. As printing systems and resins designed with curing wavelength below 405 nm continue to develop, access to 3DP fluorophore-doped material with excitations below 450 nm (e.g., protoporphyrin IX or other porphyrin-based molecules) might be attainable. Further characterization of photopolymerization effects on fluorescence emission, irradiation-induced photobleaching, and the effects of heating from high fluence rate systems on printed materials are also warranted.

The work presented lays the foundation for the manufacturing and characterization of 3DP fluorescent materials with tunable optical properties. The use of these methodologies could enable the broad adoption of fluorescent imaging targets and development of new imaging indications by addressing the shortcomings of previous manufacturing techniques in production scalability, tuning of application-specific optical spectra, and long-term stability.

## Methods

### Preparation of 3D printing resin

Preparation of the 3DP material was done with four commercially available resins: eSun Standard Clear, 3DM Clear Tough, NextGen 3Dresyn TD90, and PR48 (Colorado Photopolymer Solutions) with 3%/weight added LT1 photoinitiator (Resyner Technologies S.L). The eSun Standard Clear resin was used for the 3DP ICG-equivalent material (Fig. 2). Pre-suspended DMSO solutions of additives (fluorophores, absorbers, and scatterers) were manufactured through sonication (5 min) and subsequent stirring (2 min) to ensure homogeneous dispersion of the particles. The stock solutions were added to the base resin and stirred (~ 2 min) until a homogeneous solution was achieved. The concentration of the stock solutions and the amount added to the base resin were varied to achieve the desired concentrations in the prepared photopolymer mixture.

### 3D printing procedure

3D printing was done using a Phrozen Sonic Mini LCD MSLA 3D printer (measured irradiance of 1.1 mW/cm^2^) alongside the open-source software CHITUBOX (Version 1.6.5) to generate the layer-by-layer printing files. A layer height of 0.05 mm was used for all the printed structures, where the single layer exposure time was adjusted (between 4 and 12 s) to achieve printability in the modified resins. The 3DP material was cleaned in a 99% isopropyl bath with manual agitation for 30 s. The printed structures were then allowed to dry before being post-cured for 1 h (48 h when indicated) using a custom 405 nm lightbox with a measured irradiance of 220 μW/cm^2^. Printing supports, rafts, and skirts were added as necessary to ensure adhesion of the 3DP structures to the build plate through the entire printing process.

### Fluorescence imaging

Fluorescence imaging was performed on the PerkinElmer Solaris system. The four imaging channels of the system use the following excitation and emission detection wavelengths (1) 470 nm channel uses 470 nm excitation and detects emission in the 517–523 nm range, (2) 660 nm channel uses 660 nm excitation and detects emission in the 692–742 nm range, (3) 750 nm channel uses 735 nm excitation and detects emission in the 770–809 nm range, and (4) the 800 nm channel uses 785 nm excitation and detects wavelengths longer than 823 nm. Error bars in Fig. 2e indicate ± one standard deviation from the mean based on the region-of-interest selected in the respective fluorescence image.

### Preparation of samples for optical characterization

Fluorescence and transmission spectra were measured using 3DP solid cuvettes (12 × 12 × 40 mm^3^) that were printed to match the sample dimensions of the spectrometers. The printed samples were cleaned and post-cured for 1 h (48 h if indicated). The samples were prepared for optical measurement by subsequent polishing using 2000 grit and 3000 grit wet sanding followed by liquid polishing using a 2-step mixture (Novus 7100).

### Absorbance spectra acquisition

Absorbance spectra for the prepared 3DP cuvette samples were measured using a Varian Cary 50B UV–VIS spectrophotometer. Transmission measurements are reported by using the equation $T = {10}^{-a}$ where $T$ is the transmission and $a$ is the measured absorbance.

### Fluorescence spectra acquisition

Fluorophore-doped 3DP cuvettes using eSun Standard Clear resin were prepared for measurement of fluorescence spectral shift and emission yield change. The fluorescence spectra were obtained by subtracting the measured spectra of the 3DP base resin cuvettes from the fluorophore-doped cuvette measurements. Reference spectra and relative yield measurements were performed on DMSO solutions of the fluorophores (except for disodium fluorescein, which was measured in a H_2_O solution). Spectral feature shifts and relative fluorescence emission yield changes were calculated on the maxima of the collected absorption and emission spectra. Spectral measurements were acquired on a Horiba Fluoromax 4 fluorospectrometer. The same acquisition parameters (excitation and emission collection) are used for a given fluorophore in both DMSO/H_2_O and the 3DP material. The prepared concentrations for the fluorophores were selected to optimize for signal-to-noise and reflect typically encountered concentrations: 1000 nM for IR-125, 10 nM for the cadmium-based quantum dots, 10 μM for methylene blue, 10 μM for the rhodamine 590, and 10 μM for disodium fluorescein.

### Photostability measurements

Photostability measurements were performed on IR-125 doped 3DP cuvettes that were imaged over a 2-month period using the Li-Cor Odyssey Clx imaging system (800 nm channel, 785 nm excitation, ~ 10 mW/cm^2^). The samples were post-cured for 1 h after printing and stored in a black box at room temperature between measurements.

### Absorption and reduced scattering coefficient measurement

3DP blocks (50 × 50 × 20 mm^2^) using eSun Standard Clear resin were manufactured at various concentrations of nigrosine and TiO_2_. These blocks were prepared for imaging using 2000 grit wet sanding to reduce specular reflections. Absorption and reduced scattering coefficients were quantified using a validated reflectance geometry spatial frequency domain imaging system and software (Reflect RS, Modulim, Irvine, CA, USA). Optical properties were quantified at each of 8 wavelengths (471, 526, 591, 621, 659, 691, 731, and 851 nm) using 5 spatial projection frequencies (0.00, 0.05, 0.10, 0.15, and 0.20 mm^−1^). Error bars in the graphs indicate ± one standard deviation from the mean based on the region-of-interest selected for each of the imaged 3DP blocks.

## Supplementary Information

Supplementary Information.

Supplementary Information.

## Data Availability

The data that support the findings of this study are available from the corresponding author upon reasonable request.
