# Supplementary material for: 3D printing fluorescent material with tunable optical properties
Source: Sci Rep. 2021 Aug 24;11:17135. doi: 10.1038/s41598-021-96496-0 (PMC8384872; doi:10.1038/s41598-021-96496-0)
Supplement: Supplementary file 1 — Supplementary Information. [file 41598_2021_96496_MOESM1_ESM.docx]

**­­­ Supplementary Information**

**Article Title:** 3D printing fluorescent material with tunable optical properties

**Authors:** Alberto J. Ruiz^1,^**^2^**, Sadhya Garg^1^, Samuel Streeter^1^, Mia K. Giallorenzi^1^, Ethan P.M. LaRochelle^2^, Kimberley S. Samkoe^1^, Brian W. Pogue^1,2^

^1^Thayer School of Engineering at Dartmouth, Hanover NH 03755

^2^QUEL Imaging LLC, White River Junction VT 05001


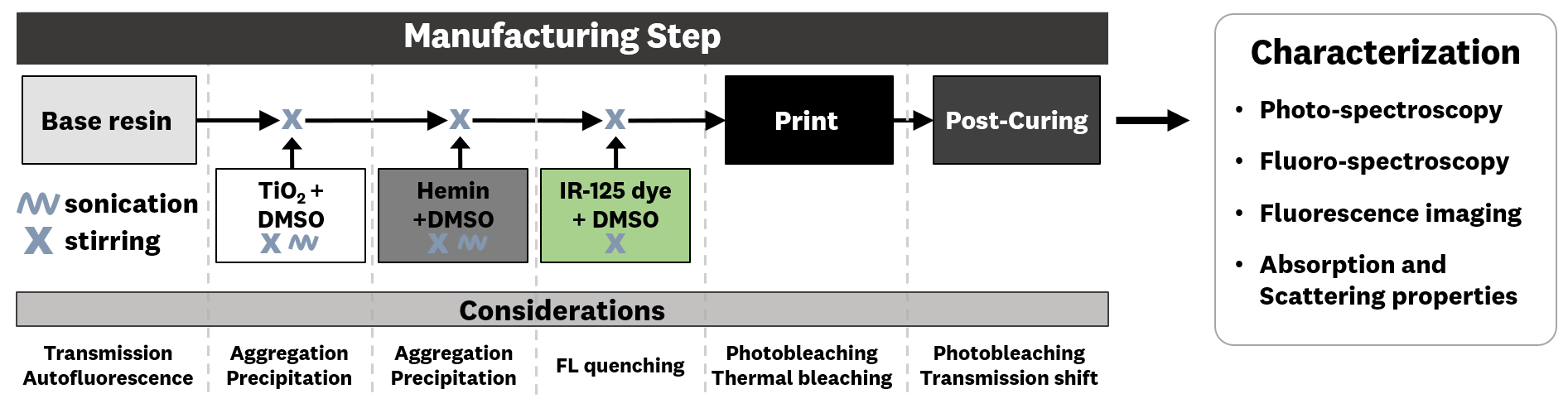


**Supplementary Fig. 1** **Manufacturing steps and respective considerations for the presented 3D printing methodology.** Pre-dispersed solutions of the fluorophores, absorbers, and scatterers are integrated into the resin to generate 3D printed fluorescent structures with tuned optical absorption and reduced scattering properties.


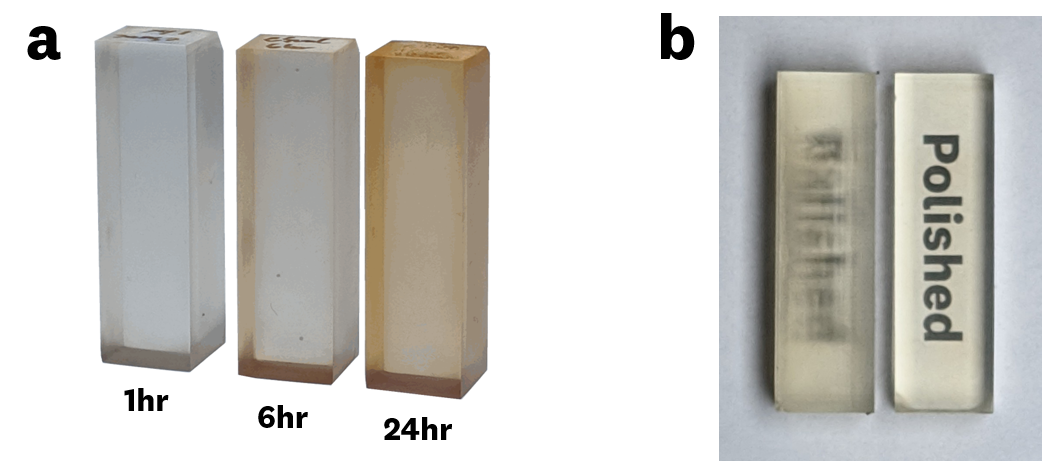


**Supplementary Fig. 2** **3DP cuvettes used for optical characterization** **a** Visualization of “yellowing” associated with increased post-curing time on 3DP solid cuvettes (eSun clear resin). **b** Polished and unpolished 3DP cuvettes. Polishing enables spectral measurements of the printed material.


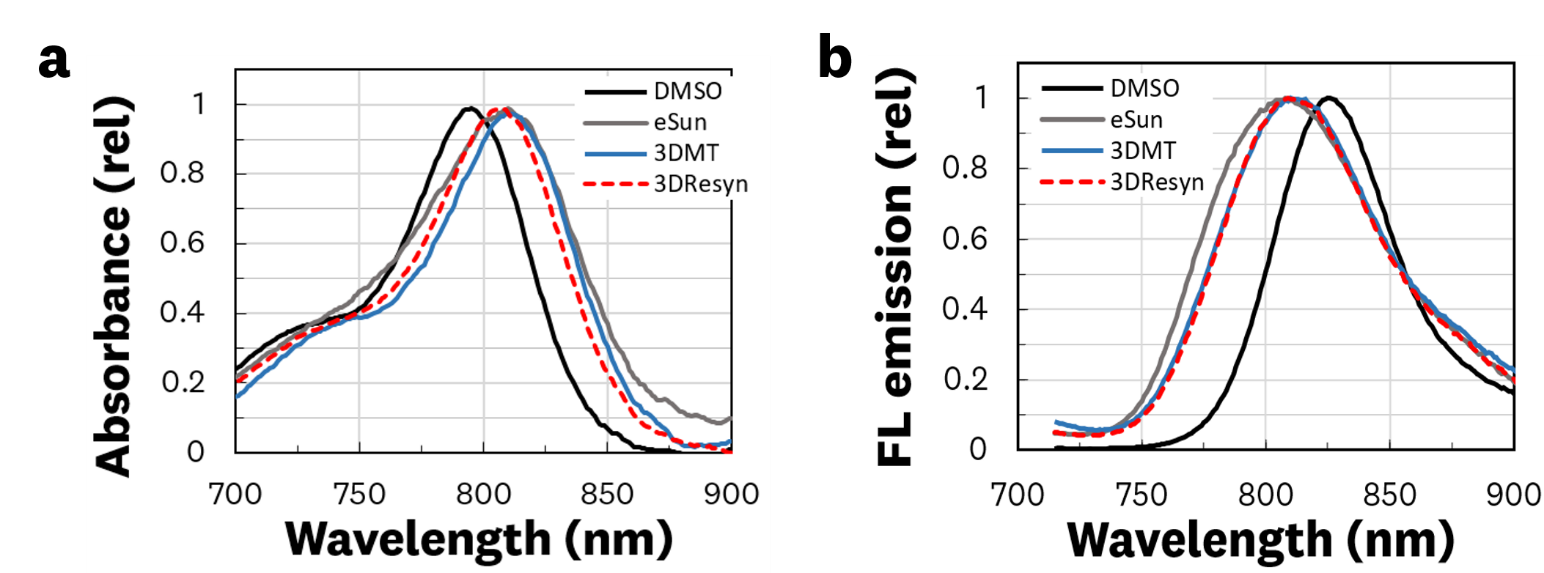


**Supplementary Fig. 3 Spectral shifts of IR-125 laser dye in three commercially available resins. a** normalized absorbance spectra **b** normalized fluorescence emission spectra. Optical measurements were performed on 3DP cuvettes with an IR-125 dye concentration of 1000nM.


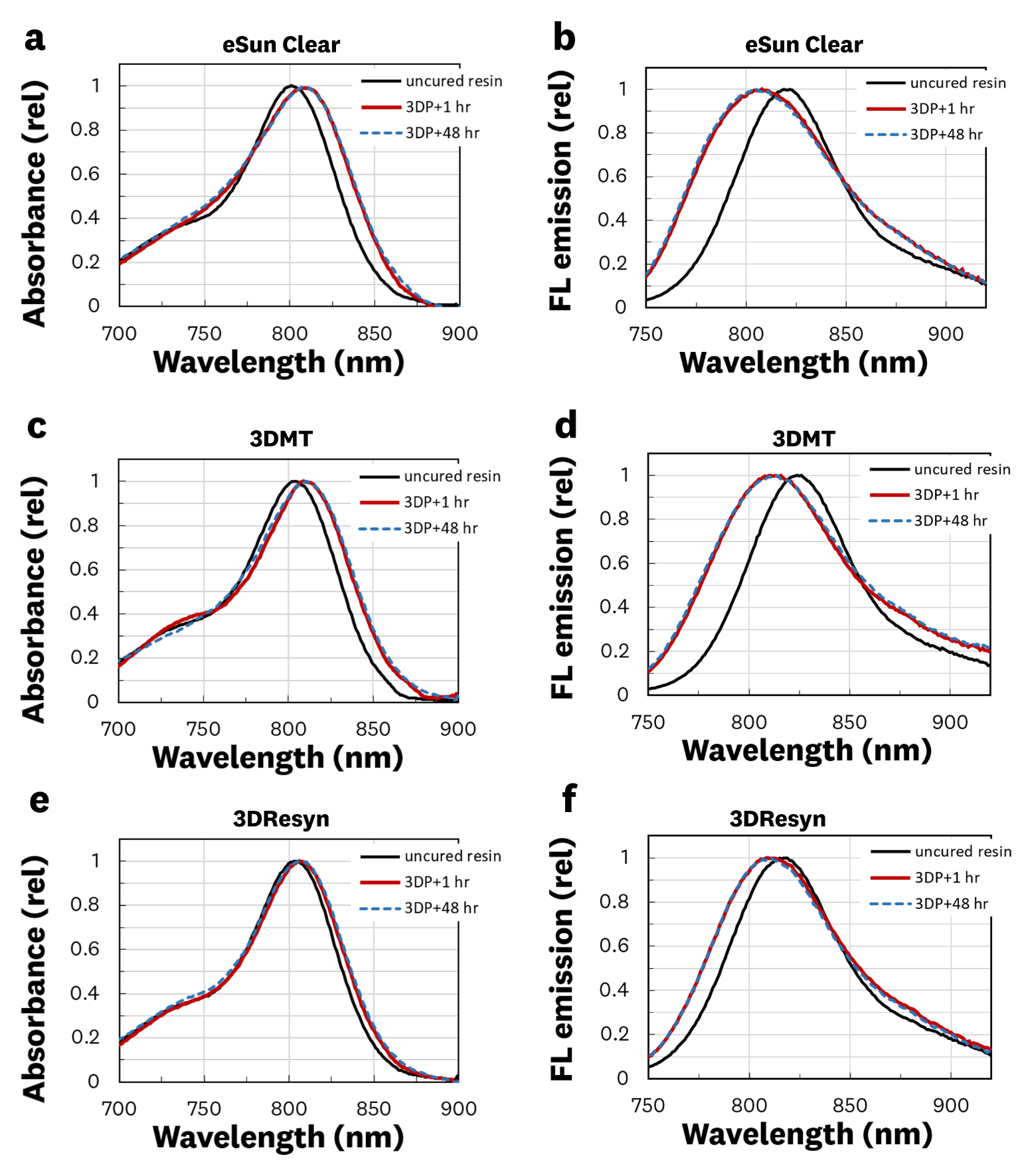


**Supplementary Fig. 4 Spectral comparison of IR-125 doped uncured resin and 3D printed material using three commercially available resins. a**,**b** eSun Standard Clear resin. **c,d** 3DM Tough resin. **e,f** , NextGen 3Dresyn TD90. Optical measurements were performed on 3DP cuvettes with an IR-125 dye concentration of 1000nM.


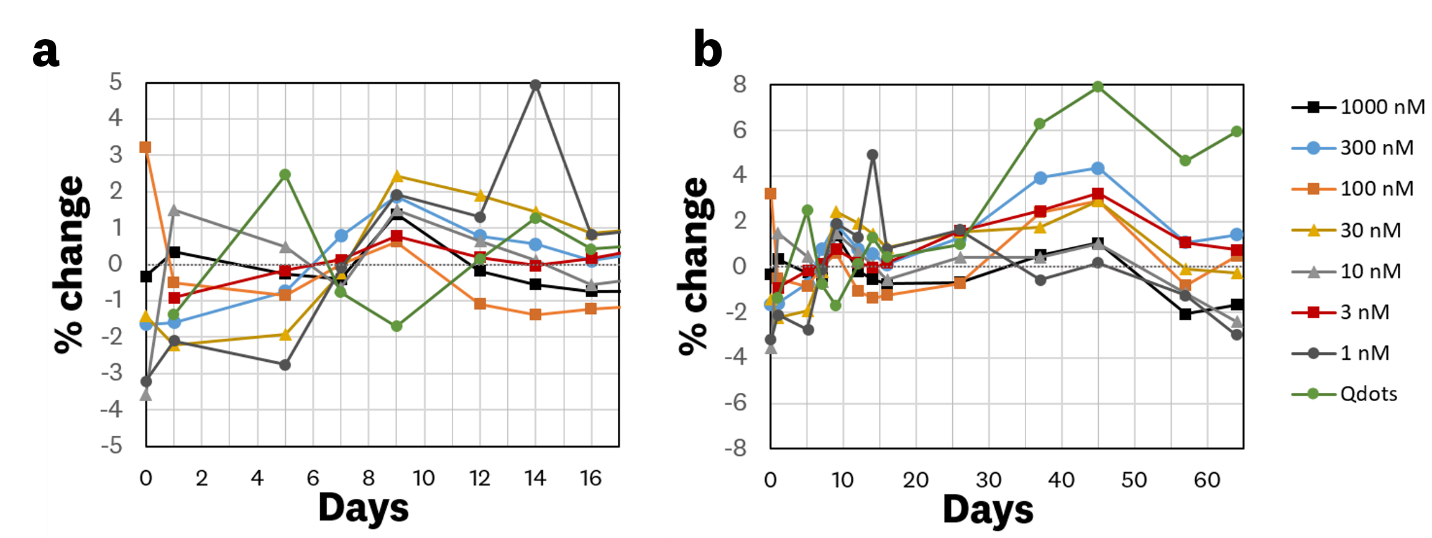


**Supplementary Fig. 5 Photostability measurements performed over two months on IR-125 and quantum dot doped 3DP material. a** First 16 days of measurements  **b** 64 days of photostability measurements. The measured standard deviation for the each timepoint is 1-2%; error bars are omitted for visual clarity.
